# The Association Between Posttraumatic Stress and Cigarette Smoking From an Ethnicity and Gender Perspective: Findings From a Longitudinal Study of U.S. Adolescents

**DOI:** 10.1002/smi.70191

**Published:** 2026-06-18

**Authors:** Johan Isaksson, Mary Schwab‐Seligman, Vladislav Ruchkin

**Affiliations:** ^1^ Department of Medical Science Child and Adolescent Psychiatry Unit Uppsala University Uppsala Sweden; ^2^ Department of Women's and Children's Health Center of Neurodevelopmental Disorders (KIND) Centre for Psychiatry Research Karolinska Institutet and Stockholm Health Care Services Region Stockholm Sweden; ^3^ Child Study Center Yale School of Medicine New Haven Connecticut USA; ^4^ Washington University School of Law St. Louis Missouri USA; ^5^ Sala Forensic Psychiatric Clinic Sala Sweden

**Keywords:** adolescents, depression, ethnicity, gender, inner‐city, longitudinal, posttraumatic stress, smoking

## Abstract

Smoking represents a significant public health problem and exploring the factors that may influence its development in youth is of major importance. The research on adolescent populations that addresses the impact of posttraumatic stress on smoking over time, especially using ethnicity and gender perspectives, remains limited. The study was conducted on a representative sample of predominantly ethnic minority youth (*N* = 2596; 53.5% female; age 11–16 years old (M(SD) = 12.75(1.27)); 58.8% African‐American, 25.5% Hispanic American, 13.6% White). Self‐reported information was obtained on smoking, symptoms of posttraumatic stress and depression in year 1 and on smoking in year 2. Generalised Linear Models were used to explore the association between posttraumatic stress in year 1 and smoking in year two in youth from the different ethnic groups, while controlling for their baseline levels of smoking, depressive symptoms, age and socio‐economic status. Posttraumatic stress was related to smoking one year later, and the association remained significant while also adjusting for the baseline variables. Sensitivity analyses indicated that the association was primarily driven by smoking frequency rather than smoking quantity. The association between posttraumatic stress and smoking did not differ by gender or ethnicity, although more females than males reported smoking both at baseline and one year later, and fewer African American adolescents reported smoking, as compared to White and Hispanic American adolescents. The findings emphasize the importance of the timely recognition of traumatic exposure and may inform targeted prevention and early interventions.

## 1 Introduction

Smoking is a critical public health issue and is regarded as the leading cause of preventable death in the United States, associated with diseases such as lung cancer, chronic obstructive pulmonary disease, and cardiovascular diseases (CDCTobaccoFree 2022). Initiation of smoking often occurs during adolescence (National Center for Chronic Disease Prevention and Health Promotion (US) Office on Smoking and Health 2014), a developmental period characterised by significant biological, psychological, and social changes. The addictive nature of nicotine is particularly concerning in the adolescent brain, which is still undergoing crucial development, and nicotine has been suggested to sensitise the brain to other drugs and prime it for future substance abuse (Yuan et al. 2015). Adolescents may also face unique vulnerabilities that increase the risk of smoking initiation and maintenance. These includes heightened sensitivity to peer influence, increased exposure to psychosocial stressors, and ongoing identity formation, which may contribute to smoking as a means of coping or social integration (Poole et al. 2022). These developmental vulnerabilities may place certain groups of adolescents at increased risk for early initiation, more persistent smoking patterns, and progression to heavier use, with ethnic minority adolescents potentially facing additional vulnerabilities. At the same time, the social consequences of adolescent smoking are also significant, with increased risk for being stigmatised for smoking resulting in stress, shame and defensiveness (Poole et al. 2022), and more likely to engage in other risky behaviours, such as illicit drug use and alcohol use (Ahun et al. 2020), which can aggregate the negative health outcomes. Given these detrimental outcomes, there is an imperative to identify the factors leading to higher risk of sustained and heavier smoking in order to find windows of opportunity for intervening at an early stage.

### 1.1 Smoking and Posttraumatic Stress

Several factors have been associated with adolescent cigarette smoking, including older age, being male, being Caucasian, lower parental education and parental smoking, lower academic performance, stress, baseline cigarette use, friends' smoking, alcohol use, and cannabis use (Ahun et al. 2020; Holliday and Gould 2016). Recent work has also focused on the influence of psychiatric symptoms for smoking in adolescence. One symptom construct that has received an increasing amount of attention is posttraumatic stress disorder (PTSD), a condition that results from traumatic exposure, and can lead individuals to engage in maladaptive coping mechanisms, one of the most prevalent being smoking. Research suggests that smokers are approximately twice more likely to have PTSD than nonsmokers, and individuals with PTSD approximately twice as likely to be current smokers (Kearns et al. 2018), to be more highly nicotine dependent and less likely to abstain from tobacco even when provided evidence‐based treatments than individuals without trauma (Shevorykin et al. 2024).

Although there is a growing body of research highlighting the association between posttraumatic stress and smoking in adolescents, only a limited number of studies have implemented longitudinal designs to examine the temporal sequencing of this relationship, and to delineate the role of posttraumatic stress from other comorbid psychological conditions that may affect smoking habits in adolescents (Kearns et al. 2018; Shevorykin et al. 2024). This may be an important oversight, as addressing these issues may provide information useful for prevention and intervention efforts. As to the temporal sequencing of comorbidity between smoking and posttraumatic stress, findings in adult populations have been mixed, indicating both that traumatic events often precede the onset of daily smoking (Forbes et al. 2015) and that in more than half of the individuals with PTSD‐smoking comorbidity regular smoking had begun prior to the development of PTSD (Cougle et al. 2010). Regarding comorbid conditions, psychiatric comorbidity is common in adolescent cigarette smokers (Upadhyaya et al. 2002) and particularly depression/depressive symptoms have been associated with smoking in adolescence (Ahun et al. 2020). Studies with adolescents on the aspects of the relationship between PTSD symptoms and smoking seldom take depression into consideration. Reviews including adult populations indicate that the PTSD–smoking relation remains even after adjusting for depression (Feldner et al. 2007; Fu et al. 2007). In addition, study populations in the previous literature on smoking and posttraumatic stress often lack diversity, with a significant proportion of research focussing on adults, particularly male veterans, and Caucasians (Shevorykin et al. 2024), thereby limiting generalisability to other groups, such as females, other ethnical groups, or younger populations.

### 1.2 Gender Differences in Smoking and Posttraumatic Stress

While previous research has demonstrated that more females than males develop posttraumatic stress after a traumatic event (Alisic et al. 2014), as well as higher prevalences of anxiety‐, trauma‐ and stress‐related disorders in females (Garza and Jovanovic 2017; McLean et al. 2011), research on smoking has reported a higher prevalence (Ma et al. 2021) and an earlier initiation (Liu et al. 2022; Okoli et al. 2013) in adolescent males, as compared to females. The extent to which the association between posttraumatic stress and smoking in adolescents varies by gender remains unclear. In a study among veterans, gender was found to moderate the association between posttraumatic stress and tobacco use, with posttraumatic stress predicting tobacco use for women, but not for men (Gross et al. 2020).

### 1.3 Ethnic Differences in Smoking and Posttraumatic Stress

Research indicates that both smoking behaviour and posttraumatic stress vary across ethnic groups, potentially reflecting differences in exposure to stressors, socioeconomic conditions, and cultural factors. Ethnic minority populations are often disproportionately exposed to trauma, including community violence, discrimination, and socioeconomic adversity (Roberts et al. 2011). For example, in Western industrialised countries, African American youths more often witness and are victims of community violence than White youths (Roberts et al. 2011; Sheats et al. 2018), and higher rates of PTSD have been reported among African American as well as Hispanic populations (Asnaani and Hall‐Clark 2017). At the same time, smoking prevalence and patterns differ across ethnic groups, and interestingly, being Caucasian has been associated with membership in trajectory groups with higher cigarette consumption (Ahun et al. 2020), while others have reported that Hispanic adolescents have been reported to be more susceptible to smoking throughout adolescence (Kamke et al. 2020). These differences highlight the importance of examining whether associations between posttraumatic stress and smoking vary across ethnic groups.

### 1.4 Socio‐Economic Status, Smoking and Posttraumatic Stress

Previous literature indicates that children and adolescents from a lower socio‐economic status (SES) backgrounds face an elevated risk for more unhealthy behaviours, including exposure to smoking during childhood, and an early initiation to smoking (Gautam et al. 2023). Similarly, adolescents from disadvantaged backgrounds are more likely to experience anxiety, depressive symptoms and emotional distress, which often co‐occur with smoking trajectories over time (Green et al. 2013), and a lower SES is associated with more severe posttraumatic stress as well as poorer treatment response (Delgadillo and Richardson 2025). These findings indicate that SES is an important contextual factor in studies of adolescent mental health and related behaviours.

### 1.5 Current Study

Within the frame of a prospective research programme, following a large cohort of U.S. inner‐city predominantly ethnic minority adolescents, we saw an opportunity to address these research questions. Specifically, this study aimed to: (i) investigate the association between posttraumatic stress and future smoking after a 1‐year interval, (ii) assess whether this association remained after adjustment for baseline smoking, SES, and depressive symptoms, and (iii) evaluate whether the patterns of associations differed by gender or ethnicity. We hypothesised that (1) posttraumatic stress in year 1 would predict smoking on a regular basis one year later, (2) the association would remain significant even after adjusting for depressive symptoms, SES and smoking at baseline, and (3) the association between posttraumatic stress and smoking may differ by ethnicity and gender.

## 2 Methods

### 2.1 Participants

This longitudinal study included adolescents aged 11–16 enroled in the public school system of New Haven, Connecticut, a city located in the northeast of the US with 134,000 inhabitants. A large proportion of the city's residents belongs to ethnic minority groups with a low socioeconomic status. At baseline, 3575 students completed the first survey. At the time for the follow‐up survey one year later, 768 of the students had left the public school system and were therefore excluded from this study. Of the remaining 2807 students, 211 lacked a complete data set with 61 students missing data on smoking at year 1 and 128 at year 2, 36 students missing data on posttraumatic stress and 40 students on depressive symptoms. Missing data was completely missing at random (Little's MCAR test Chi‐square = 17.88; *p* = 0.657), why a complete‐case analysis was used given the large sample size. The final sample (*N* = 2596, 53.5% female, mean age in year one 12.75 (SD = 1.27)), was predominantly comprised of minority ethnicities (58.8% African‐American, 25.5% Hispanic American, 13.6% White, 2.0% Other), an accurate reflection of the local public‐school population (New Haven Public Schools, 2019). The sample population was predominantly socio‐economically disadvantaged, as reflected by the large proportion (over 71.7%), who qualified for free/reduced lunch status at either point of the data collection.

### 2.2 Procedure

Parents were informed of the survey at the time of school registration, received a letter about the survey 2 weeks prior to its administration, and were offered the opportunity to decline participation. The passive informed consent procedure was approved by the university's institutional review board and considered as an appropriate ethical procedure by the state legislature. Prior to the survey's administration, students were read a detailed assent form outlining their participation with the assurance of confidentiality and were asked to sign it to indicate assent (parent and child refusals were less than 1%). Students completed the survey in their classrooms during a single class period on a regular school day. Trained administrators read all the questions aloud, while students followed along with their copies of the survey, reading questions to themselves and circling responses in the booklet. A second administrator was available, providing help to individual students on request. Surveys were administered in English and Spanish. All students in the respective grades attending schools on the day of the survey's administration were eligible to participate. Make‐up administrations were performed at each school within 1 month of the initial administration for those who had been absent.

### 2.3 Measures

#### 2.3.1 Smoking

In years 1 and 2 smoking was assessed with two items: “during the past 30 days, how many days did you smoke” and “during the past 30 days, how many cigarettes did you smoke per day?”. The students responded on a four‐point Likert scale. For the first question the response items were 0 = I did not smoke, 1 = A few days, 2 = Most days, 3 = every day; and for the second question 0 = I did not smoke, 1 = One cigarette per day, 2 = Two to five cigarettes per day, 3 = Six or more cigarettes per day. The two items were summed because they reflected closely related aspects of recent smoking involvement, were strongly correlated (*ρ* = 0.80 at year 1, and *ρ* = 0.85 at year 2) and had a large proportion of non‐smokers. The measure had a possible total score ranging between 0 and 6, with a higher score indicating more smoking during the last month.

#### 2.3.2 Posttraumatic Stress

Posttraumatic stress was assessed in year 1 with the Child Post‐Traumatic Stress—Reaction Index (CPTS‐RI) (Pynoos et al. 1987). This measure has been widely used in previous studies to assess symptoms of posttraumatic stress, for example, in children and adolescents after a traumatic exposure (Pynoos et al. 1987). CPTS‐RI consists of 20 items assessing the frequency of posttraumatic stress symptoms the last four weeks on a 5‐point scale, ranging from “Never” (0) to “Most of the time” (4). The total score could range from 0 to 80, with higher scores corresponding closely with a clinical diagnosis of PTSD (Steinberg et al. 2004)). McDonald's omega was 0.89.

#### 2.3.3 Depressive Symptoms

In year 1 Depressive symptoms were assessed using an adaptation of the Centre for Epidemiologic Studies‐Depression Scale (CES‐D) (Radloff 1977), which has demonstrated excellent psychometric properties with adolescent populations (Carpenter et al. 1998). The scale consists of 10 statements (e.g., “I felt like crying”; “I felt that many bad things were my fault”), inquiring about the presence of depressive symptoms during the past month, which are assessed using a three‐point scale (“Not true” (0); “Somewhat true” (1); or “Certainly true” (2)). The total score can range from 0 to 20, with higher scores indicating more depressive symptoms. McDonald's omega was 0.82.

#### 2.3.4 Socioeconomic Status

Information about students' eligibility for subsidised school lunch were collected. Data were obtained from the school register, with three possible price options: full price (scored as 0), reduced price (scored 1) or free lunch (scored 2). This information was then used as a proxy for socioeconomic status (SES), with higher scores indicating worse SES. At the time of the data collection, students whose family income was 185% or less of the federal poverty line were eligible for free or reduced lunches. The use of information about the students' eligibility for subsidised school lunches as a proxy measure for SES has occurred in previous studies, for example, (Ruchkin et al. 2023).

### 2.4 Statistical Analyses

Data were analysed using SPSS version 32. Possible gender differences in the study variables were assessed using a Mann‐Whitney U‐tests, and differences by race were investigated with Kruskal Wallis test. The normality of the outcome variable (smoking year two) distribution was explored. The data showed a right‐skewed gamma distribution and hence were not normally distributed. The data were treated as count data and the Akaike information criterion indicated that a negative binomial distribution with log link had the best model fit. Therefore, Generalised Linear Models (GLM) with negative binomial log link were used for the main analyses. First, we investigated the association between posttraumatic stress in year one and smoking in year two, also adjusting for gender, age, SES, and ethnicity (dummy variables for African American and Hispanic American students). In the next model we additionally adjusted for smoking in year one, and in the final model we additionally adjusted for symptoms of depression. Interaction effects between posttraumatic stress by gender and ethnicity on future smoking was tested in the fully adjusted model. Further, as sensitivity analyses, we re‐calculated the fully adjusted model for the two items on smoking separately, that is, number of smoking days and number of cigarettes per day. A *p*‐value < 0.05 was considered significant.

## 3 Results

### 3.1 Characteristics of the Sample and Group Comparisons

The mean values of all the study variables are presented in Table 1. In year one 88.2%, and in year two 85.1% reported not having smoked any cigarettes last month. In year one, 7.0%, 1.9% and 1.0% reported having smoked a few days, most days and every day respectively. The corresponding numbers for year two were 7.5%, 2.9% and 2.6%. As to the number of cigarettes, in year one 6.7% reported smoking one cigarette a day, 2.6%—two to five cigarettes a day and 0.8%—six or more cigarettes a day. For year two the corresponding numbers were 6.7%, 4.8% and 1.8%. As shown in Table 1, more females than males reported smoking in year one and year two. Furthermore, females reported more posttraumatic stress and depressive symptoms in year one than males.

**TABLE 1:** Gender comparisons of the study variables (Mean (SD)) Study Variables with Mann‐Whitney *U*‐test.

	Total sample	Girls *n* = 1388	Boys *n* = 1208	Statistics
Posttraumatic stress year 1	21.81 (13.08)	23.56 (13.42)	19.81 (12.38)	*Z* = 7.68^a^
Age	12.75 (1.27)	12.73 (1.30)	12.77 (1.24)	*Z* = 1.23
SES	1.35 (0.89)	1.35 (0.90)	1.36 (0.89)	*Z* = 0.44
Smoking year 1	0.28 (0.89)	0.31 (0.91)	0.25 (0.85)	*Z* = 2.35^b^
Smoking year 2	0.43 (1.18)	0.47 (1.17)	0.38 (1.19)	*Z* = 3.54^a^
Depressive symptoms	4.67 (4.19)	5.50 (4.49)	3.72 (3.61)	*Z* = 10.44^a^

Abbreviations: M, Mean; SD, Standard deviation, SES, Socioeconomic status.

^a^ *p* < 0.001.

^b^ *p* < 0.05.

The mean values of reported smoking in year 1 and 2 by ethnicity are presented in Table 2. There were ethnic differences in smoking in year one (H (3) = 9.51, *p* = 0.023), smoking in year two (H (3) = 23.18, *p* < 0.001), and in posttraumatic stress (H (3) = 12.07, *p* = 0.007). More specifically, African American students smoked less in year one and two compared to both White (*p* = 0.037 and *p* < 0.001, respectively) and Hispanic American students (*p* = 0.008 and *p* = 0.001, respectively). Further, White students had lower ratings on posttraumatic stress compared to African American (*p* < 0.001) and Hispanic American (*p* = 0.030) students.

**TABLE 2:** Smoking year 1 and 2, and posttraumatic stress, stratified by ethnicity.

	Caucasian	African American	Hispanic	Other
Smoking year 1 M (SD); Median rank	0.44 (1.24); 1330.26	0.23 (0.77); 1278.55	0.33 (0.93); 1330.10	0.19 (0.65); 1265.45
Smoking year 2 M (SD); Median rank	0.75 (1.64); 1380.28	0.33 (1.03); 1264.66	0.48 (1.19); 1334.72	0.36 (1.09); 1273.59
Posttraumatic stress M (SD); Median rank	20.46 (14.02); 1180.79	22.18 (12.80); 1331.93	21.79 (13.34); 1287.64	20.43 (10.81); 1258.13

Abbreviations: M, Mean; SD, Standard deviation.

### 3.2 Longitudinal Association Between Posttraumatic Stress and Smoking

The findings are presented in Table 3. Posttraumatic stress was related to smoking one year later, and the association remained significant even in the fully adjusted model, although attenuated. There was an effect for age and gender, with higher levels of smoking among older students and females, but the association between female gender and smoking became non‐significant when adjusting for depressive symptoms. Smoking in year one predicted smoking one year later. Further, being African‐American was associated with less smoking. SES and depressive symptoms were not related to future smoking. No interaction effect of gender or race by posttraumatic stress on smoking one year later was found.

**TABLE 3:** Smoking in year two regressed on posttraumatic stress, smoking, demographic data and symptoms of depression in year one.

	Model 1			Model 2			Model 3		
	Beta	95% CI	*p*‐value	Beta	95% CI	*p*‐value	Beta	95% CI	*p*‐value
Posttraumatic stress year 1	**0.023**	**0.017, 0.028**	**< 0.001**	**0.014**	**0.008, 0.020**	**< 0.001**	**0.010**	**0.002, 0.017**	**0.013**
Gender (female)	**0.162**	**0.013, 0.311**	**0.033**	**0.159**	**0.002, 0.317**	**0.048**	0.137	−0.023, 0.297	0.094
Age	**0.329**	**0.272, 0.386**	**< 0.001**	**0.185**	**0.123 0.247**	**< 0.001**	**0.175**	**0.111, 0.238**	**< 0.001**
SES	0.004	−0.084, 0.093	0.927	0.035	−0.059, 0.128	0.469	0.035	−0.059, 0.128	0.470
African American	**−0.638**	**−0.837, −0.439**	**< 0.001**	**−0.469**	**−0.708, −0.279**	**< 0.001**	**−0.489**	**−0.703, −0.274**	**< 0.001**
Hispanic	−0.222	−0.449, 0.005	0.055	−0.172	−0.416, 0.071	0.166	−0.177	−0.421, 0.067	0.154
Smoking year 1				**0.543**	**0.479, 0.608**	**< 0.001**	**0.544**	**0.480, 0.609**	**< 0.001**
Depressive symptoms							0.020	−0.003, 0.044	0.085

*Note:* Bold indicates *p* < 0.05.

Abbreviation: SES, Socioeconomic status.

### 3.3 Sensitivity Analyses on the Longitudinal Association Between Posttraumatic Stress and Number of Days Smoking and Number of Cigarettes

As shown in Table S1 and Table S2, posttraumatic stress was related to number of days of smoking the last 30 days across the models, whereas the association between posttraumatic stress and number of cigarettes per day was only significant in the first model when not adjusting for number of cigarettes and depressive symptoms at year 1. Number of days smoking and number of cigarettes predicted the same outcomes one year later.

## 4 Discussion

This study evaluated the longitudinal associations between posttraumatic stress symptoms and smoking in a large general population sample of inner‐city, predominantly ethnic minority U.S. adolescents. In this study, posttraumatic stress was related to smoking one year later, and the association remained significant when adjusted for symptoms of depression and smoking at baseline. The sensitivity analyses indicated that the association was primarily driven by number of days smoking rather than number of cigarettes a day. The association between posttraumatic stress and future smoking did not differ by gender or ethnicity. Interestingly, more females reported smoking at both baseline and one year later. Fewer African‐American students reported smoking, compared to Caucasian and Hispanic‐American students.

In line with our hypothesis, posttraumatic stress predicted smoking on a regular basis one year later. This corroborates the previous finding of an association between PTSD and smoking (Kearns et al. 2018; Shevorykin et al. 2024) in which traumatic events preceded the onset of daily smoking among adults (Forbes et al. 2015). PTSD symptoms and anxiety can lead individuals to engage in maladaptive coping mechanisms, as expressed in the self‐medication (Khantzian 1997) and tension‐reduction (Kalodner et al. 1989; Thayer et al. 1994) theories explaining the link between PTSD and substance use. The self‐medication hypothesis posits that individuals use substances such as nicotine to regulate distressing internal states, including intrusive memories, hyperarousal, and negative affect, which are central features of posttraumatic stress. Complementarily, tension‐reduction theory suggests that substance use is negatively reinforced through its capacity to reduce physiological arousal and emotional tension, thereby increasing the likelihood of repeated use. Smoking may therefore be used by those with posttraumatic stress to alleviate symptoms. Importantly, our finding that posttraumatic stress was associated with future smoking frequency rather than quantity may further refine this theoretical interpretation. It suggests that adolescents may engage in smoking as a repeated, situation‐dependent coping strategy to manage ongoing distress, rather than escalating consumption per occasion. Consistent with this interpretation, a recent review of factors associated with tobacco use identified regulation of trauma‐related negative affect as a common explanatory mechanism (Shevorykin et al. 2024).

Our large community‐based sample was comprised of participants mainly in early to middle adolescence, with approximately 7% reporting having smoked in the last month and 2.5% reported smoking every day. This is of importance from a public health perspective given the potential risk for nicotine dependence among adolescent smokers (Yuan et al. 2015), and for other risky behaviours, including illicit drug and alcohol use (Ahun et al. 2020). Even though psychiatric comorbidity (Upadhyaya et al. 2002), and especially depressive symptoms (Ahun et al. 2020), are common in adolescent cigarette smokers, the association between posttraumatic stress and smoking in our study remained significant even after adjusting for symptoms of depression, and depressive symptoms were not associated with future smoking in the fully adjusted model. Although, research implementing longitudinal designs to explore the association between posttraumatic stress and smoking in adolescent populations are sparse, our findings support findings from adult populations where the association between PTSD and smoking similarly remained significant even when adjusted for depression (Feldner et al. 2007; Fu et al. 2007). At the same time, because this was an observational study, the findings should be interpreted as showing a prospective association rather than a causal or uniquely determining effect.

White and Hispanic American students, reported more smoking than African‐American students, as expected from the literature (Ahun et al. 2020). The reported lower rates of smoking among African‐American students have been previously linked to such factors as a strong parental discipline, limited financial resources for buying tobacco products, and an emphasis on religious and family values in these communities (Beech and Scarinci 2003). More females than males reported smoking, a finding that seemingly contrasts with the impression that adolescent males have a higher cigarette consumption than their female peers (Ahun et al. 2020) and that males have a lower age of smoking initiation than females (Okoli et al. 2013). However, in another review on the gender differences in smoking, females were reported to be more likely to smoke but were less likely than males to become heavy smokers (Rodham et al. 2005). Gender differences in smoking across adolescence are complex, and likely to be context‐dependent and shaped by social norms and structural conditions. For instance, economic context has been suggested to influence gender differences in initiation, with girls in lower‐income settings sometimes reporting earlier smoking than boys (Liu et al. 2022). An additional explanation for these seemingly contradictory findings may lie in how gender prescriptions and proscriptions shape adolescents' engagement with smoking. Bottorff et al. (2014) suggested that gender roles, identities, and relations shape tobacco use and cessation, with smoking carrying culturally specific meanings related to femininity and masculinity. In the present study, however, these should be understood as interpretive possibilities rather than directly tested mechanisms.

Our findings contribute to the literature by examining gender and ethnic‐specificity in the relation between posttraumatic stress and smoking, and suggest that this association may be relatively consistent across these groups rather than being specific to any one subgroup. This indicates that posttraumatic stress may represent a general risk factor for smoking across adolescents, regardless of gender or ethnic background. Although there is a shortage of studies investigating whether ethnicity or gender moderate this relationship during adolescence, our findings do not support strong moderating effects and thus contrast with some prior research in adult samples. For example, one study found that the relationship between PTSD and smoking was stronger among White compared to Black individuals (Wilson et al. 2014), while another study among veterans reported that posttraumatic stress predicted tobacco use in women but not in men (Gross et al. 2020). Such discrepancies may reflect developmental differences, sample characteristics, contextual influences, or limited power to detect interaction effects, and further research in adolescent populations is needed.

Although prior research shows that lower SES is linked to greater exposure to smoking, earlier initiation, and co‐occurring mental health difficulties (Gautam et al. 2023; Green et al. 2013; Delgadillo et al., 2025), SES was not associated with future smoking in our models‐ This may suggest that SES‐related disparities may operate differently in this sample or that other factors such as ethnicity, baseline smoking, and posttraumatic stress played a more prominent role in explaining later smoking involvement. It is also possible that SES effects are more pronounced for smoking initiation than for continuation, or that SES differences were attenuated in a sample with relatively high overall socioeconomic disadvantage and low smoking prevalence.

### 4.1 Strengths and Limitations

The strengths of this study include the data collection from a large sample of school youth, the longitudinal study design, and a close collaboration with the local public school system which allowed for involvement of nearly all youth of their school system in order to learn and serve them more effectively. There are several limitations to be noted. This study utilised self‐report data, which can be subject to reporting bias, although the research team assured anonymity and no identifying personal information was not asked on the questionnaire. Use of other sources of data, for example from parents and teachers, would have improved confidence in our findings; however, doing so was not feasible. The study was limited to a group of mainly economically deprived American inner‐city youth, and no claim is made as to whether the results can be generalised to other adolescent populations. We had some missing data, which potentially could have biased estimates of the correlations reported, but we strongly doubt that this was of a large enough magnitude to override the consistency of data that led to our team's conclusions. Some data were missing, but at random. Since fewer than 5% of participants had missing data on any of the study variables, and the research team had a relatively large sample that could detect small effect sizes, a complete‐case analysis was used for dealing with missing data, as suggested by previous literature (Lodder 2014; Pedersen et al. 2017). Other factors that we could not assess might potentially have been important for the observed associations, such as parental smoking or substance use, parental psychopathology, peer smoking, access to cigarettes, and trauma type (Ahun et al. 2020; Stone et al. 2012). Also, other psychological variables relevant to maladaptive coping such as sense of control and stress‐management skills were not included in the models, despite their central role in theories of self‐medication and tension‐reduction mechanisms. Lastly, any inferences on causality cannot be made because of the correlational nature of the study.

## 5 Conclusions

Posttraumatic stress symptoms were associated with later smoking during early and middle adolescence, even after accounting for SES, smoking and symptoms of depression at baseline. The sensitivity analyses suggested that the prospective association with the composite smoking score was primarily attributable to smoking frequency rather than smoking quantity. The association between posttraumatic stress and smoking was similar for males and females, and did not differ by ethnicity. The findings align with theories considering smoking as a form of self‐medication and tension‐reduction and emphasise the importance of timely recognition of traumatic exposure that may potentially be important for deterring possible detrimental outcomes related to mental health issues as well as inform targeted prevention and early intervention efforts. Future studies should employ longitudinal designs over a longer period across adolescence and young adulthood to explore the association between posttraumatic stress and smoking, also including other sources of data besides self‐reports, as well as data on any provided treatment for posttraumatic stress and additional psychosocial and contextual variables to further clarify these associations.

## Ethics Statement

This study was approved by the Institutional Review Board at the Yale School of Medicine. Informed consent was obtained and information was read and provided in written form to all students in the consent process. Information about the purpose, survey, and procedure was provided to parents along with access to information about how to refuse the child/youth's participation prior to data collection. This study was initiated as an evaluation tool and information source for the Social Development Programme of New Haven Public Schools (NHPS).

## Conflicts of Interest

The authors declare no conflicts of interest.

## Supporting information


**Table S1:** Number of days smoking in year two regressed on posttraumatic stress, number of days smoking, demographic data and symptoms of depression in year one.

**Table S2:** Number of cigarettes per day in year two regressed on posttraumatic stress, number of cigarettes, demographic data and symptoms of depression in year one.

## Data Availability

The datasets used in this article are not publicly available because the data cannot be shared except in publicly reported form due to the initial decision of the local ethics committees, as well as the restrictions included in the informed consent statement where it was stated that the data would only be used by the research group and would not in other than aggregated form be utilized elsewhere. Access to the datasets is not permitted except to authorized members of the New Haven Public Schools Social Development evaluation team, and concerns should be directed to the authors.
