# Supplementary material for: The Association Between Posttraumatic Stress and Cigarette Smoking From an Ethnicity and Gender Perspective: Findings From a Longitudinal Study of U.S. Adolescents
Source: Stress Health. 2026 Jun 18;42(3):e70191. doi: 10.1002/smi.70191 (PMC13277632; doi:10.1002/smi.70191)
Supplement: Supplementary file 1 — Table S1: Number of days smoking in year two regressed on posttraumatic stress, number of days smoking, demographic data and symptoms of depression in year one. [file SMI-42-e70191-s002.docx]

| **SUPPLEMENTARY TABLE 1** Number of days smoking in year two regressed on posttraumatic stress, number of days smoking, demographic data and symptoms of depression in year one |
| --- |

|  | Model 1 | | | Model 2 | | | Model 3 | | |
| --- | --- | --- | --- | --- | --- | --- | --- | --- | --- |
|  | beta | 95% CI | p-value | beta | 95% CI | p-value | beta | 95% CI | p-value |
| Posttraumatic stress year 1 | **.023** | **.016, .029** | **<.001** | **.014** | **.006, .021** | **<.001** | **.011** | **.001, .021** | **.024** |
| Gender (female) | .075 | -.119, .268 | .449 | .068 | -.136, .272 | .315 | .054 | -.153, .260 | .610 |
| Age | **.350** | **.277, .423** | **<.001** | **.223** | **.144 .303** | **<.001** | **.217** | **.136, .298** | **<.001** |
| SES | .014 | -.102, .129 | .818 | .035 | -.087, .156 | .313 | .036 | -.086, .157 | .567 |
| African American | **-.648** | **-.905, -.392** | **<.001** | **-.475** | **-.750, -.201** | **<.001** | **-.471** | **-.747, -.196** | **<.001** |
| Hispanic | -.186 | -.477, .105 | .210 | -.121 | -.189, .432 | .444 | -.125 | -.436, .186 | .431 |
| Days smoking year 1 |  |  |  | **.906** | **.768, 1.044** | **<.001** | **.906** | **.768, 1.044** | **<.001** |
| Depressive symptoms |  |  |  |  |  |  | .013 | -.017, .042 | .407 |

SES, Socioeconomic status

Bold indicate p < 0.05
