# Supplementary material for: The Association Between Posttraumatic Stress and Cigarette Smoking From an Ethnicity and Gender Perspective: Findings From a Longitudinal Study of U.S. Adolescents
Source: Stress Health. 2026 Jun 18;42(3):e70191. doi: 10.1002/smi.70191 (PMC13277632; doi:10.1002/smi.70191)
Supplement: Supplementary file 2 — Table S2: Number of cigarettes per day in year two regressed on posttraumatic stress, number of cigarettes, demographic data and symptoms of depression in year one. [file SMI-42-e70191-s001.docx]

| **SUPPLEMENTARY TABLE 2** Number of cigarettes per day in year two regressed on posttraumatic stress, number of cigarettes, demographic data and symptoms of depression in year one |
| --- |

|  | Model 1 | | | Model 2 | | | Model 3 | | |
| --- | --- | --- | --- | --- | --- | --- | --- | --- | --- |
|  | beta | 95% CI | p-value | beta | 95% CI | p-value | beta | 95% CI | p-value |
| Posttraumatic stress year 1 | **.004** | **.000, .008** | **.031** | .002 | -.002, .006 | .273 | .001 | -.004, .007 | .651 |
| Gender (female) | .037 | -.069, .143 | .449 | .033 | .074, -.140 | .545 | .027 | -.136, .081 | .620 |
| Age | **.057** | **.015, .098** | **.007** | .026 | -.017 .069 | .235 | .024 | -.020, .067 | .283 |
| SES | -.001 | -.064, .063 | .981 | .001 | -.063, .065 | .975 | .001 | -.063, .065 | .974 |
| African American | -.139 | -.292, .015 | .077 | -.105 | -.261, .050 | .183 | -.104 | -.259, .051 | .190 |
| Hispanic | -.078 | -.255, .099 | .388 | -.065 | -.243, .113 | .475 | -.066 | -.244, .113 | .470 |
| Nr of cigarettes year 1 |  |  |  | **.317** | **.211, .422** | **<.001** | **.317** | **.212, .422** | **<.001** |
| Depressive symptoms |  |  |  |  |  |  | .005 | -.011, .022 | .536 |

SES, Socioeconomic status

Bold indicate p < 0.05
